# Supplementary material for: Evolution of cardiac tissue and flow mechanics in developing Japanese Medaka
Source: PLoS One. 2024 Aug 26;19(8):e0309018. doi: 10.1371/journal.pone.0309018 (PMC11346936; doi:10.1371/journal.pone.0309018)
Supplement: S1 Fig — Each instantaneous image sequence is divided into windows and the same windows for two consecutive images are used for processing. A 50% Gaussian filtering is applied on each window and the filtered windows are cross-correlated using SCC to get instantaneous shift estimates. This process is followed for image pairs in the same phase for different cycles. The SCC planes from different cycles are averaged to obtain an ensemble SCC plane. A subpixel fit is done to estimate the peak location which denotes the shift between image pairs. This process is repeated for all windows in the image. An outlier detection is performed on the vector field. The whole process is repeated for multi-pass processing with the estimated field used to deform the original image windows. (PDF) [file pone.0309018.s001.pdf]

Processing methodology

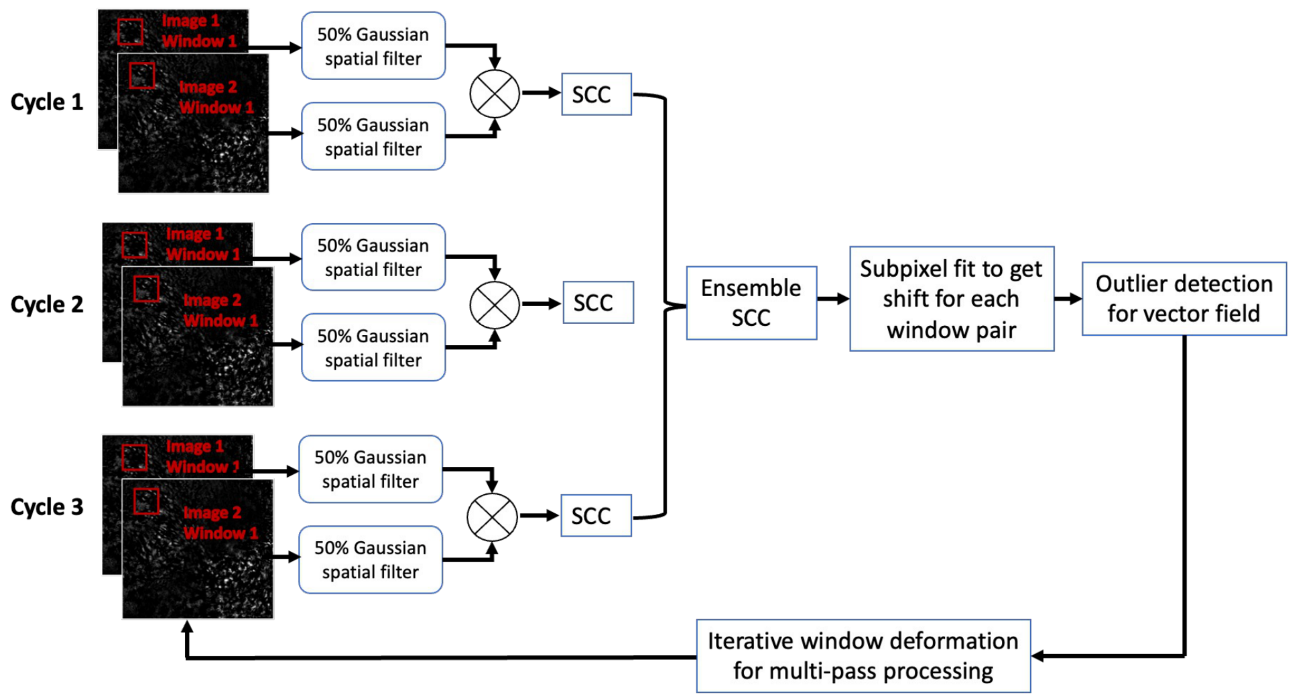

S1 Fig: A schematic of the PIV image processing steps. Each instantaneous image sequence is divided into windows and the same windows for two consecutive images are used for processing. A 50% Gaussian filtering is applied on each window and the filtered windows are cross-correlated using SCC to get instantaneous shift estimates. This process is followed for image pairs in the same phase for different cycles. The SCC planes from different cycles are averaged to obtain an ensemble SCC plane. A subpixel fit is done to estimate the peak location which denotes the shift between image pairs. This process is repeated for all windows in the image. An outlier detection is performed on the vector field. The whole process is repeated for multi-pass processing with the estimated field used to deform the original image windows.
