# Supplementary material for: Evolution of cardiac tissue and flow mechanics in developing Japanese Medaka
Source: PLoS One. 2024 Aug 26;19(8):e0309018. doi: 10.1371/journal.pone.0309018 (PMC11346936; doi:10.1371/journal.pone.0309018)
Supplement: S1 Table — The table compares Peak Straindias, peak ΔPAVC, peak ΔPOFT, peak ΔPAVCHR*μ, Peak ΔPOFTHR*μ and EW between each pair of dpfs. n = 5 at each dpf. *** denotes p<0.0001. ** denotes p<0.001. * denotes p<0.05. (PDF) [file pone.0309018.s002.pdf]

Statistical analysis

Statistical significance was tested for all the cardiac function and geometry parameters using Tukey-Kramer Honest Significant Difference (HSD) test. The p-values that denote the difference of mean values of the parameters (peak strain rate at ventricle diastole, dimensional and non-dimensional peak pressure drop at AVC and OFT, endocardial work) between a pair of dpf are listed in S1 Table.

S1 Table: p-values from Tukey Kramer HSD tests. The table compares Peak $Strain_{dias}$, peak ΔP_{AVC}, peak ΔP_{OFT}, peak $\frac{\Delta P_{AVC}}{HR*\mu}$, Peak $\frac{\Delta P_{OFT}}{HR*\mu}$ and EW between each pair of dpfs. n=5 at each dpf. *** denotes $p<0.0001$. ** denotes $p<0.001$. * denotes $p<0.05$.

**p-values**							
**dpf**		**Peak $Strain_{dias}$**	**Peak ΔP_{AVC}**	**Peak ΔP_{OFT}**	**Peak $\frac{\Delta P_{AVC}}{HR*\mu}$**	**Peak $\frac{\Delta P_{OFT}}{HR*\mu}$**	**EW**
3	4	1.0	1.0	1.0	1.0	1.0	1.0
3	5	0.0303*	1.0	1.0	1.0	1.0	0.9858
3	6	0.5798	0.6091	1.0	0.2685	1.0	0.9258
3	7	0.0168*	0.8229	1.0	0.8459	1.0	0.28
3	8	0.1596	0.3828	1.0	0.4985	1.0	0.7129
3	9	<0.0001***	0.0004**	0.3816	0.0026*	0.9496	<0.0001***
3	10	0.0072*	0.0018*	0.0403*	0.0044*	0.0172*	0.0001**
3	11	0.0016*	0.0458*	0.0633	0.3054	0.3856	0.1152
3	12	<0.0001***	0.0008**	0.9067	0.0041*	0.9946	<0.0001***
3	13	<0.0001***	0.0327*	0.1715	0.4106	0.8427	<0.0001***
3	14	0.0414*	0.0088*	0.141	0.2771	0.8272	0.0077*

4	5	0.0289*	1.0	1.0	1.0	1.0	0.9875
4	6	0.5671	0.5836	0.9999	0.2099	0.9984	0.9321
4	7	0.0159*	0.8028	0.9999	0.7771	1.0	0.29
4	8	0.1538	0.3633	1.0	0.4255	1.0	0.7244
4	9	<0.0001***	0.0004**	0.2725	0.0018*	0.7787	<0.0001***
4	10	0.0069*	0.0016*	0.0275*	0.0029*	0.007*	0.0001**
4	11	0.0015*	0.0418*	0.0427*	0.2447	0.1875	0.12
4	12	<0.0001***	0.0007**	0.7847	0.0027*	0.914	<0.0001***
4	13	<0.0001***	0.0295*	0.1157	0.3337	0.5699	<0.0001***
4	14	0.0394*	0.008*	0.0955	0.2203	0.5791	0.0082*
5	6	0.9023	0.9352	1.0	0.6741	0.9993	1.0
5	7	1.0	0.9894	1.0	0.9918	1.0	0.9586
5	8	0.9999	0.7437	1.0	0.8364	1.0	0.9994
5	9	0.4924	0.0051*	0.2853	0.0235*	0.7975	<0.0001***
5	10	1.0	0.0176*	0.0239*	0.0358*	0.005*	0.0099*
5	11	0.998	0.2067	0.0384*	0.6919	0.174	0.7383
5	12	0.5874	0.0091*	0.8172	0.0338*	0.9276	<0.0001***
5	13	0.5969	0.1771	0.1116	0.8174	0.5731	<0.0001***
5	14	1.0	0.0573	0.096	0.6579	0.5919	0.2099
6	7	0.8651	1.0	1.0	0.9979	1.0	0.9825
6	8	0.9988	1.0	1.0	1.0	1.0	0.9999
6	9	0.0077*	0.1566	0.7532	0.7971	0.9991	<0.0001***
6	10	0.7039	0.3659	0.2505	0.879	0.1742	0.0099*

6	11	0.3118	0.9367	0.3308	1.0	0.863	0.8092
6	12	0.0118*	0.2377	0.9966	0.8691	1.0	<0.0001***
6	13	0.0124*	0.9339	0.5741	1.0	0.9954	<0.0001***
6	14	0.9669	0.6308	0.44	1.0	0.9884	0.2436
7	8	0.9999	0.9982	1.0	0.9996	1.0	1.0
7	9	0.4004	0.0710	0.7469	0.2249	0.9865	0.0001**
7	10	1.0	0.1948	0.2452	0.3062	0.0806	0.3026
7	11	0.9968	0.8005	0.3244	0.9973	0.6619	1.0
7	12	0.4966	0.1154	0.9963	0.294	0.9994	0.0038*
7	13	0.5066	0.7839	0.5662	0.9999	0.9562	0.0009**
7	14	1.0	0.4136	0.4334	0.9957	0.9347	0.9493
8	9	0.1453	0.7349	0.6883	0.895	0.9481	0.0001**
8	10	0.9978	0.9226	0.2007	0.942	0.0419*	0.1829
8	11	0.8973	0.9999	0.2704	1.0	0.482	0.996
8	12	0.195	0.8357	0.9915	0.9366	0.993	0.0026*
8	13	0.2006	0.9999	0.4962	1.0	0.8683	0.0007**
8	14	1.0	0.9831	0.3761	1.0	0.8414	0.8133
9	10	0.5924	1.0	1.0	1.0	0.7213	0.1144
9	11	0.9755	0.9739	1.0	0.8852	0.9999	0.0036*
9	12	1.0	1.0	0.9919	1.0	1.0	0.9988
9	13	1.0	0.9422	1.0	0.6395	1.0	1.0
9	14	0.2241	0.9999	1.0	0.9062	1.0	0.0110*
10	11	0.9998	0.9991	1.0	0.9388	0.9499	0.8369

10	12	0.6919	1.0	0.6934	1.0	0.205	0.6635
10	13	0.7015	0.9967	0.9999	0.7473	0.5776	0.3483
10	14	1.0	1.0	1.0	0.9524	0.8913	0.9923
11	12	0.9899	0.9927	0.8028	0.9327	0.9537	0.0515
11	13	0.9909	1.0	1.0	1.0	0.9998	0.0168*
11	14	0.9716	1.0	1.0	1.0	1.0	0.9999
12	13	1.0	0.9805	0.9651	0.7332	0.9999	1.0
12	14	0.2957	1.0	0.8737	0.9473	0.9993	0.1496
13	14	0.3036	0.9999	1.0	1.0	1.0	0.0514
