# Supplementary material for: Evolution of cardiac tissue and flow mechanics in developing Japanese Medaka
Source: PLoS One. 2024 Aug 26;19(8):e0309018. doi: 10.1371/journal.pone.0309018 (PMC11346936; doi:10.1371/journal.pone.0309018)
Supplement: Attachment — Submitted filename: Response to Reviewers.docx [file pone.0309018.s003.docx]

**Response to the reviewer’s comments for the article: “Evolution of cardiac tissue and flow mechanics in developing Japanese Medaka”**

**Reviewer #1:**

The manuscript "Evolution of cardiac tissue and flow mechanics in developing Japanese Medaka” uses image analysis of image sequences obtained with high speed cameras to calculate blood flow and pressure in the developing medaka heart. The presented data provide interesting parameter analysis during embryonic heart development. The manuscript is of mainly descriptive nature reporting a specialized methodology without reaching novel conclusions on heart development or function. The authors should provide a conclusion why their results are relevant for a study of embryonic heart development, and explain how their work goes beyond being mostly descriptive. It would be good to provide an outlook on how they envision that their methodology will contribute to an analysis and understanding of heart development and function. Nevertheless this work is of interest for the wide spectrum of readers of PlosOne and thus merits publication after some revision.

Answer: We thank the reviewer for the comments and feedback on the quality of the manuscript. A detailed step-by-step response is given below with changes highlighted by an underline.

The following sentences (lines 535 - 538) have been added in the conclusion to highlight the relevance of results in embryonic heart development -

“Overall, a consistent increase in pressure drop, and higher EW during embryonic heart development is a measure of narrowing AVC and increasing wall contractility associated with cardiac remodeling. The EW can also be correlated with the metabolic demand of the heart. A deviation in the measured metric could indicate abnormal heart development.”

To explain how the methodology contributes to an analysis and understanding of heart development, the following lines (lines 538 - 542) are added in the conclusion.

“The automated advanced boundary segmentation and flow processing algorithm increases the reliability of the measured metrics. The non-invasive hydrodynamic measurements estimated using our novel workflow can be further developed as a diagnostic tool to differentiate between normal and diseased hearts.”

minor points line 91: The authors should specify the strain used for this study. Several highly inbred strains are available for medaka. Use of an inbred strain allows to keep variance during analysis low which is important for acquisition of quantitative data.

Answer: The medaka strain information has been added as follows – “Wild type adult Japanese medaka of an inbred SK2MC strain (20 male-female pairs) obtained from USEPA, Duluth, MN, USA”

line 92: please give details what “continuous oxygen supply” means. Does this refer to continuous water circulation?

Answer: Yes, the phrase has been changed to “continuous oxygen supplied by an aquarium pump”.

An additional sentence has been added describing the levels - “Dissolved oxygen level was always above 5 mg/L, pH was between 7–8, and total ammonia levels were below 0.25 ppm.”

line 100: were any measures taken to improve transparency of the chorion or rather reduce chorion induced image distortions when imaging embryos prior to hatching?

Answer: This is clarified in lines 99-100 as follows – “Chorions were intact with the embryos and image distortions were assumed to be negligible due to the presence of chorions.”

lines 103 ff: the imaging methodology of unhatched and hatched embryos/hatchling has to be described better: in which medium were the embryos immersed during imaging? Was a climate chamber used to keep T constant? Were hatchlings anesthetized during the imaging to prevent moving? How was an imaging of a lateral view achieved in the actively swimming hatchlings?

Answer: The following clarification has been added in the fish selection for imaging subsection (lines 102-106) –

Line 103: “The temperature was kept constant by placing the samples in a chamber whose temperature was between 26$^{\circ}$C and 28$^{\circ}$C.”

Line 105: “Larvae were anesthetized using MS-222 (226 mg/L) solution to prevent movement during imaging.”

line 150 the terms AVC and OFT need an introduction here for the understanding

Answer: The terms AVC and OFT are defined earlier in line 80 of the manuscript. However, the authors have expanded the abbreviation in line 150 for ease of understanding.

line 195: give a definition of hematocrit for the understanding

Answer: The hematocrit definition was added to the lines 198-199 as follows –

“Then the Ht, defined by the percentage by volume of RBC present in the blood, was calculated using equation (4)”

line 278: here is the place to use the term diastole together with “ventricle filling cycle”.

Answer: The term diastole has been added to line 279 as follows- “Pressure field variation across the atrium and ventricle along three phases of the ventricle filling cycle i.e., diastole is plotted in Fig 3”

**Reviewer #2:**

The provided manuscript presents a study of the evolution of a set of biomechanical characteristics during cardiac morphogenesis in the Japanese Medaka. Using microscopy particle image velocimetry quantifications, 2D viscous fluid velocity fields in the valve regions were reconstructed; based on which pressure fields and endocardial wall strains at several developmental time points were calculated. The authors demonstrated that pressure drops, and the relationship between pressure and strain can be used to distinguish developmental time points.

We thank the reviewer for their careful review and feedback. We have incorporated the comments and highlighted the changes in this document.

L27-L28: The authors mention that “The effects of pressure drop [...] triggering cardiac morphogenesis in a teleost heart species are poorly understood”, but they do not further consider this in their manuscript. As such, it is misleading and should be altered.

Answer: The sentence “The effects of pressure drop across cardiac valve cushion regions and endocardial wall strain triggering cardiac morphogenesis in a teleost species heart are poorly understood.” has been modified to reflect better the paper’s findings and how it addresses the literature gap – “The effects of pressure drop across cardiac valve cushion regions and endocardial wall strain in the early developmental stages of a teleost species heart are poorly understood.”

L40-L42: Missing reference(s)

Answer: The following reference has been added – “Andrés-Delgado, Laura, and Nadia Mercader. "Interplay between cardiac function and heart development." Biochimica et Biophysica Acta (BBA)-Molecular Cell Research 1863.7 (2016): 1707-1716.”

L42-L45: Missing reference(s)

Answer: Existing references cited - “However, past studies [2],[3] investigating biomechanics have mainly probed into the flow wall shear stress (WSS) variation,”

L47-L49: Missing reference(s)

Answer: Existing reference cited – “The EW has been shown in humans and higher vertebrates to directly reflect oxygen demand and metabolic consumption [4].”

L58-L59: “viscous

Answer: The reviewer’s comment is not clear in this case. The lines mention – “Pressure gradients in the zebrafish (Danio rerio) heart were calculated using Laplace law [12] or from the knowledge of circumferential stress [13]. However, the accuracy of these measurements is limited since they neglected viscous forces in the heart.” This is true as viscous resistance is neglected in reference [13].

L61-L62: “[...] because the [opacity of the] fish’s eyes and head blocked [visibility of] a part of the ventricle.”

Answer: Thank you for the clarification. This has been taken into account as follows –

“The imaging was limited to the atrium only because the opacity of fish’s eyes and head blocked visibility of a part of the ventricle.”

L95: Missing concentration of diluted saline solution

Answer: The saline solution was 50% diluted. This is added in line 97 – “50% diluted saline solution containing 2 ppm methylene blue”

L115: “RBC” - abbreviation not previously defined/introduced

Answer: This is corrected to explain the abbreviation as follows –

In L115, “fluctuations of red blood corpuscle (RBC) patterns”

L119-L122; Figure 1: Scale bars are missing in all panels

Answer: The scale bars are now shown for Fig 1 on the right-hand side as can be seen in the image below –

The following line is added in the Fig 1 caption to clarify this – “The scalebar in μm is shown on the right side.”

L124: “HR” - abbreviation not previously defined/introduced

Answer: The sentence has been corrected to include the abbreviation definition – “The heart rate (HR) was measured”

L126: Missing reference for parameter value (blood density)

Answer: A blood plasma density value of 1025 Kg/m^3^ is assumed as the blood density. This is noted from the following reference – Frcitas, R. A. (1998). Exploratory Design in Medical Nanotechnology: A Mechanical Artificial Red Cell. Artificial Cells, Blood Substitutes, and Biotechnology, 26(4), 411–430. <https://doi.org/10.3109/10731199809117682>. Currently, the phrase in line 126 reads: “blood density (ρ) as 1025 kg/m3 [21]”

L127: “EF” - abbreviation not previously defined/introduced

Answer: L133 now reads – “The ejection fraction (EF) is a function of ventricle volumes at diastole and systole.”

Equation 1: The assumption of cylindrical ventricle area and volume appears to be unjustified, as its shape deviates from tubular already one day post-fertilization (1dpf). The authors themselves show in their Figure 2b-d sketches of non-cylindrical ventricles.

Answer: Assuming axisymmetric cylindrical ventricle shape and neglecting ventricle size variation at different cross-sections is present in similar measurements (Bulk, A., Bark, D., Jr, Johnson, B., Garrity, D., & Dasi, L. P. (2016). Mechanisms influencing retrograde flow in the atrioventricular canal during early embryonic cardiogenesis. Journal of biomechanics, 49(14), 3162–3167. <https://doi.org/10.1016/j.jbiomech.2016.07.028>). However, the authors agree that the approach has limitations considering the actual 3D shape. The planar measurement in micro-PIV is limited in resolving the depth of the 3D irregularly shaped volume and area of the ventricle. We acknowledge the limitation in our assumption as follows –

“Following Bulk et al. [13], a cylindrical area and volume are assumed for the ventricle due to limitations in resolving depth in a planar micro-PIV measurement. However, the assumption is restrictive as the ventricle deviates from tubular shape during development.”

L131-L156: A visualization of the image processing steps would have been beneficial for the readers to understand the effect of the filtering.

Answer: Lines 131 to 156 describe the advanced PIV processing algorithm which involves several steps as mentioned in the cited publications. Depicting each step in detail in a figure would be difficult. However, to aid visualization of the image processing steps, a schematic of the key steps is shown in the following figure added in the supporting information.

“S2 Fig: A schematic of the PIV image processing steps showing each instantaneous image sequence is divided into windows and the same windows for two consecutive images are used for processing. A 50% Gaussian filtering is applied on each window and the filtered windows are cross-correlated using SCC to get instantaneous shift estimates. This process is followed for image pairs in the same phase for different cycles. The SCC planes from different cycles are averaged to obtain an ensemble SCC plane. A subpixel fit is done to estimate the peak location which denotes the shift between image pairs. This process is repeated for all windows in the image. An outlier detection is performed on the vector field. The whole process is repeated for multi-pass processing with the estimated field used to deform the original image windows.”

L157-L165; Figure 2: On which sources of information are the sketches based?

Answer: Figure 2a is a picture taken by the author. Figures 2b-2d are representative sketches based on the visual observations by the author during experiments.

This is clarified in the figure caption as follows – “Figures 2b-2d are representative sketches based on the author’s visual observations during experiments.”

L203: “ [...] as a function of hematocrit [percentage] (Ht[%]) [...]”

Answer: Based on reviewer 1’s comment we defined the Hematocrit as the volume percentage of RBC present in blood – “Then the Ht, defined by the percentage by volume of RBC present in the blood, was calculated using equation (4)**.**” From this definition, Hematocrit (Ht) is always referred to as a percentage and we are not assigning a “[%]” sign alongside the symbol.

Equation 7: Not referenced in the text; Δ is not introduced; the first two terms on the right-hand side of equation 6 mentioned in L220-L221 as being negligible are still present.

L226: “Writing in terms of the [dimensions] [...]”

L229; Equation 10: Should add P= M/(LTT), = M/(LT) and insert into equation 10 to show dimensions vanish.

Answer: Last three comments are taken into account and this section has been modified as follows –

“Equation (6) denotes a rearranged form of the unsteady Navier Stokes equation for incompressible flow. We denote a representative length scale, time scale and velocity scale of the flow by $L$, $T$, and $U$ respectively where $U=L/T$. To deduce the correct non-dimensionalization for pressure from equation (6), we subsitute $L, T, U, P$ and do an order of magnitude analysis of the individual terms to obtain equation (7).

$$\frac{P}{L}=\rho\left( \frac{U}{T} \right)+\rho\left( \frac{U^{2}}{L} \right)+\mu\left( \frac{U}{L^{2}} \right)$$

(7)

Multiplying by (LT/µ), we get,

$$\frac{P}{\left( \frac{1}{T} \right)*\mu}=\rho\left( \frac{UL}{\mu} \right)+\rho\left( \frac{U^{2}T}{\mu} \right)+\mu\left( \frac{U T}{L \mu} \right)$$

(8)

As $U=L/T$, the first two terms on right side of equation (8) can be simplified to Re. For creeping flow (Re<<1), thus, these terms become negligible. The third term on the right side simplifies to 1. Hence, the pressure term should be non-dimensionalized by $\left( \frac{1}{T} \right)*\mu$. Since, heart rate has dimensions $\left( \frac{1}{T} \right)$, we get the dimensionless equation for pressure as,

$$\frac{P}{HR*\mu}=1$$

(9)

Considering the dimensions of individual terms, $P=M/(LTT)$, $\mu=M/(LT)$ and $HR=1/T$, we can see that equation (9) is dimensionless.”
